# Unsupervised Learning of Depth and Camera Pose with Feature Map Warping

**DOI:** 10.3390/s21030923

**Published:** 2021-01-30

**Authors:** Ente Guo, Zhifeng Chen, Yanlin Zhou, Dapeng Oliver Wu

**Affiliations:** 1 College of Physics and Information Engineering, Fuzhou University, Fuzhou 350108, China; guoente@gmail.com; 2 Department of Electrical and Computer Engineering, University of Florida, Gainesville, FL 32611, USA; zhou.y@ufl.edu (Y.Z.); dpwu@ieee.org (D.O.W.)

**Keywords:** monocular depth estimation, single camera egomotion, occlusion-aware mask network, feature pyramid matching loss

## Abstract

Estimating the depth of image and egomotion of agent are important for autonomous and robot in understanding the surrounding environment and avoiding collision. Most existing unsupervised methods estimate depth and camera egomotion by minimizing photometric error between adjacent frames. However, the photometric consistency sometimes does not meet the real situation, such as brightness change, moving objects and occlusion. To reduce the influence of brightness change, we propose a feature pyramid matching loss (FPML) which captures the trainable feature error between a current and the adjacent frames and therefore it is more robust than photometric error. In addition, we propose the occlusion-aware mask (OAM) network which can indicate occlusion according to change of masks to improve estimation accuracy of depth and camera pose. The experimental results verify that the proposed unsupervised approach is highly competitive against the state-of-the-art methods, both qualitatively and quantitatively. Specifically, our method reduces absolute relative error (Abs Rel) by 0.017–0.088.

## 1. Introduction

Vision-based environment depth and egomotion estimation are essential for autonomous vehicle perception and infrastructure-less robot navigation [1]. At present, LiDAR and RGB-D cameras have been widely used in the depth measurement. LiDAR has become more precise and cheaper, such as Livox mid-40, 100, but it is still not perfect, like the small field of view, irregular scanning pattern, nonrepetitive scanning and motion blur [2]. The application of RGB-D cameras in outdoor environments has also become more extensive, but the measurement range is limited [3]. Therefore, in order to deal with the complex outdoor environment, real outdoor robotic applications focus on multiple sensor fusion. In this context, the better each individual sensor is, the better the final result is [4]. The monocular is attractive because it has the advantages of low price, high resolution, rich information acquisition. More accurate monocular depth estimation is helpful for depth estimation of multiple sensor fusion. Therefore, obtaining depth based on monocular is a valuable study. Recent deep learning-based methods have shown great success on monocular depth and egomotion estimation [5,6]. These methods can be divided into two categories: supervised learning methods [5,7,8] and unsupervised learning methods [9,10,11,12,13,14,15,16,17,18]. Our work focuses on monocular unsupervised method of depth and egomotion estimation, since supervised method requires time-consuming handicraft labels.

Most unsupervised learning methods estimate depth and camera egomotion by minimizing a photometric error [10]. The photometric error is the sum of absolute differences (SAD) between the warped frame and target frame, where the warped frame is obtained from adjacent one, predicted depth and relative camera motion of the target frame [9,10]. A common assumption used by current works is photometric consistency, that is, the photometric error of corresponding pixel of the same object in different frames is zero. The photometric consistency assumption is often not satisfied because of brightness change and non-Lambertian surface [19]. To overcome these issues, GeoNet [11] added structural similarity (SSIM) [20] to loss to mitigate the effects of brightness change. SSIM captures more local information than SAD, but it does not capture global information. D3VO [19] predicted the global transformation parameters a,b through a network, and adjusts the image *I* to aI+b. However, D3VO only pays attention to the global brightness change, which is often hard to be satisfied in the real scene. None of these methods consider both local and global information.

In addition, the dynamic objects and occlusion also violate the photometric consistency. To overcome the problem of dynamic objects, the unsupervised method struct2depth [13] segmented all objects in the image and then estimated the 3D motion of each object. This method is suitable for highly dynamic scenes, but the accuracy of the depth is affected by 3D motion estimation. Furthermore, SC-SfmLearner [6] proposed a self-discovery mask for handling moving objects, which improves the accuracy of depth estimation. However, its mask definition adopts relative error, and thus is not sensitive to depth changes in areas with large depth, which causes inaccurate depth estimation. Regarding the occlusion problem, as far as we know, there is no existing unsupervised method in literature.

Our contributions are as follows.

1. We propose feature pyramid matching loss (FPML) capturing local and global information, which is more robust than SAD and SSIM and can solve the problem of photometric inconsistency caused by brightness change.

2. The proposed occlusion-aware mask (OAM) addresses, for the first time, the problem of photometric inconsistency causing by occluded pixels in the image with the consideration of novel relationship between two adjacent masks.

3. Furthermore, OAM solves the problem of dynamic objects by balancing the photometric error and the regularization term of the mask and improve the accuracy of depth and camera egomotion.

## 2. Related Work

The development of deep learning has facilitated the application of supervised and unsupervised methods. We briefly overview some supervised depth estimation methods and introduce current SOTA unsupervised methods for single view depth and egomotion estimation.

### 2.1. Supervised Depth Estimation Via Convolutional Neural Network (CNN)

The supervised learning methods establish the relationship between image and corresponding depth through CNN. Eigen et al. [7] first proposed using CNN to predict monocular image depth in 2014. They proposed a multiscale method that uses two deep network stacks: one makes a rough global prediction based on the whole image, and the other optimizes the prediction locally. Eigen et al. [8] improved the previous method by increasing the number of multiscale layers to obtain more image details. They used a single multiscale CNN architecture to accomplish three different computer vision tasks: depth prediction, surface normal estimation and semantic labeling. Li et al. [21] improved depth estimation on the basis of Eigen et al. [7] and proposed a fast-to-train multiscale CNN with skip connections between multiscale layers to speed up convergence during training. Laina et al. [22] proposed a fully convolutional network, encompassing residual learning to map monocular images to depth. They presented a novel upsampling method to improve the output resolution and introduced the reverse Huber loss to improve the accuracy of depth estimation. Xu et al. [23] proposed a deep model that fuses complementary information derived from multiple CNN side outputs. They presented two fusion methods: one is based on a cascade of multiple conditional random fields and the other is based on a unified graphical model.

The above-mentioned supervised methods need a large number of ground truths during training, but acquiring ground truths is difficult in practice. Using synthetic data is a good alternative, but these data cannot simulate the physical world accurately [24].

### 2.2. Unsupervised Depth and Egomotion Estimation

Compared with the supervised methods, the unsupervised learning methods do not need labels; thus, the latter methods overcome the disadvantage of the supervised learning relying on labels. Unsupervised depth and camera egomotion estimation only needs raw video sequences. These methods refines the model from the video gathered from a new scene [13]; thus, it can be rapidly deployed in practical applications.

Garg et al. [25] proposed an unsupervised depth estimation method using stereo pairs for the first time. The autoencoder network predicts the depth of the left image, and a reconstructed the left image is synthesized by epipolar geometry constraint [26] and the right image. The photometric error between the left image and the synthesized left image is used as a loss term to train the autoencoder network. Godard et al. [9] extended Garg’s work and proposed the left-right depth consistent loss function to improve the accuracy of depth estimation. Stereo unsupervised learning requires stereo image pairs and the known pose between stereo cameras during training.

SfmLearner [10] only used the monocular video sequence while learning the monocular depth and egomotion in a coupled way. They used depth network to predict monocular depth and pose network to predict the relative camera pose between consecutive frames. The color inconsistency between target image and synthesized target images, which warped from the reference image, was used as the supervision signal. SfmLearner proposed an explainability mask to alleviate the influence of moving objects and non-Lambertian surfaces for making the system more robust. SFM-Net [12] outputted k motion objects’ mask and their rigid motion through the motion network to overcome the influence of moving objects. However, it is limited by the maximum number of moving objects. In contrast to SFM-Net, Yin et al. [11] decomposed motion into rigid and nonrigid components and introduced a residual flow learning module to deal with nonrigid scenes. Casser et al. [13] segmented all possible moving objects by Mask R-CNN [27] before training and then estimated the 3D motion of each object to overcome the weakness of SFM-Net. However, masking all possible moving objects prevents the network from learning the depth object and Mask RCNN increases the amount of calculation. SC-SfMLearner [6] proposed a self-discovery mask for dynamic scene in consideration of geometric consistency constraints, which improves the accuracy of depth estimation. However, it has room for improvement in the area of large depth, because the relative error decreases with the increase of depth in the case of the same absolute error of depth. We propose OAM, which can not only address the problem of occluded pixels but also reduce the depth blur caused by moving objects.

Most of these methods are based on photometric errors and assume constant brightness and Lambertian surface of objects. However, meeting these conditions is difficult in real scenes. To handle the problem, [9,11,13,28,29] added SSIM [20] as a loss term to produce more robust matching and improve the performance of depth prediction. Unsupervised optical flow [30] also used the photometric error as loss function. They adopted robust kernel functions to deal with cases in which photometric consistency assumptions are not met. In contrast to hand-craft feature, we propose a FPML that is inspired by PWC-Net cost volume [31]. Instead of matching hand-craft features, a trainable feature pyramid is constructed by CNN.

## 3. Method

### 3.1. Preliminaries

**Figure 1:**
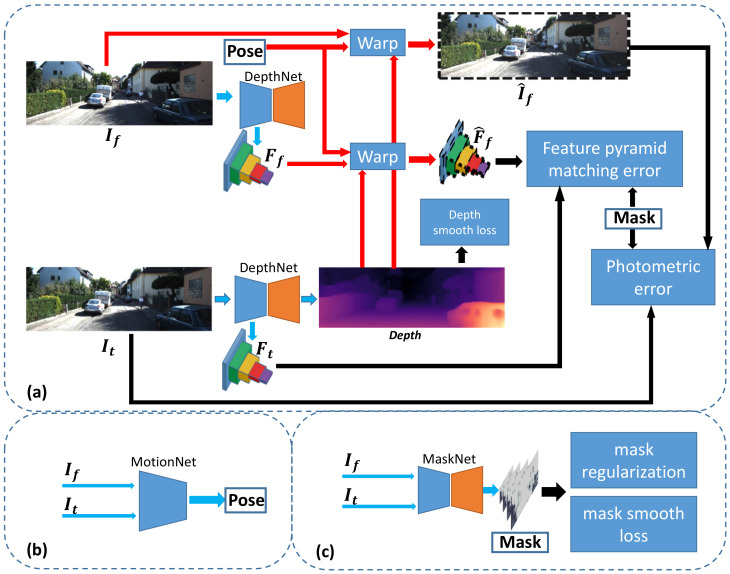
System architecture. (**a**) DepthNet, loss function and warping; (**b**) MotionNet (**c**) MaskNet. It consists of the DepthNet for predicting depth map of the current frame $I_{t}$, the MotionNet for estimating egomotion from current frame $I_{t}$ to adjacent frame $I_{f}$, and the MaskNet for generating occlusion-aware mask (OAM). The reconstructed current frame ${\hat{I}}_{f}$ and reconstructed current feature pyramid ${\hat{F}}_{f}$ are synthesized by warping. The total loss function consists of photometric error, depth smooth loss, mask regularization term, mask smooth loss and feature pyramid matching loss (FPML).

Our method uses single-view depth and multiview pose networks, with a loss based on warping the adjacent frames to the current frame using the computed depth and pose. In this work, we propose a framework containing three networks: a depth prediction network (DepthNet), a camera egomotion network (MotionNet) and an occlusion-aware mask network (MaskNet). The networks will be trained together due to the loss function but can be applied independently at test time. The framework of the networks and loss functions are shown in Figure 1, in which the blue arrows represent the input and output of the networks. DepthNet input is a frame, which can predict the corresponding depth. The information of multiple frames is enough to estimate the camera egomotion [26], so the input of MotionNet is the current frame $I_{t}$ and the adjacent frames $I_{f}$. The output of MotionNet is the camera egomotion $T_{t\to f}$, including rotation Euler angle and 3D position, where the adjacent frames include the past and next frames, $I_{f}\in \left\{I_{t-1},I_{t+1}\right\}$. In order to predict occluded pixels and moving objects, the input of MaskNet is the current frame and the adjacent frames, and the output is consistent mask $M_{f}$ and occlusion mask $V_{f}$. The masks outputted by the MaskNet are only used in the training stage. It can exclude pixels that do not conform to the static scene and are occluded, ensuring that DepthNet and MotionNet can learn the correct depth and camera egomotion respectively. In the training phase, DepthNet, MaskNet and MotionNet are trained at the same time. However, in the testing phase, MaskNet is not needed, so it can be called an auxiliary network for auxiliary training. The details of the networks are described in Section 3.5.

The warp process is to find the corresponding point in the adjacent frames through the depth map of the current frame and the camera egomotion, and then synthesize the current frame. The warping process is divided into two steps: coordinate transformation and interpolation reconstruction. According to the pinhole camera model, $P=D_{t}\left(p_{t}\right)K^{-1}p_{t}$ is a back projection process [26], where *P* represents a point in 3D space, $p_{t}$ denotes the homogeneous coordinate of the point on the current frame, *K* is the given camera intrinsic parameters, and $D_{t}\left(p_{t}\right)$ is the depth of $p_{t}$. The projection $p_{f}$ of P in the adjacent frames is inferred as follows,

(1) $$\begin{array}{cc} D_{f}\left(p_{f}\right)p_{f} & =KT_{t\to f}\left[\begin{array}{c} P\\ 1 \end{array}\right]\\ \; & =K(D_{t}\left(p_{t}\right)T_{t\to f}\left[\begin{array}{c} K^{-1}p_{t}\\ 1 \end{array}\right]). \end{array}$$

The process of interpolation reconstruction is to synthesize the pixel value of $p_{t}$ according to the adjacent frames, ${\hat{I}}_{f}\left(p_{t}\right)=I_{f}\left(p_{f}\right)$, where ${\hat{I}}_{f}$ represents the current frame synthesized by $I_{f}$. We use the differentiable bilinear interpolation proposed by the spatial transformer network [32] to obtain $I_{f}\left(p_{f}\right)=\sum_{i,j}\omega_{i,j}I_{f}\left(p_{f}^{i,j}\right)$, where $p_{f}^{i,j}$ is the integer pixel located at the neighborhood (top left, top right, bottom left, and bottom right) of $p_{f}$, and $\sum_{i,j}\omega_{i,j}=1$. As shown in Figure 1, the red arrows in the framework are the input and output of the warp module. The warp process of the feature map is similar to the warp of the RGB image, except that the multichannel feature map replaces the three-channel color.

The loss we propose includes a photometric error $L_{p}$ weighted by the OAM, a depth smoothness loss $L_{s}$, a mask regularization loss $L_{m}$, a mask smoothness loss $L_{ms}$ and the FPML $L_{f}$. we define overall loss function as follows,

(2) $$L_{all}=\sum_{n=0}^{3}\left(L_{p}^{n}+\lambda_{s}L_{s}^{n}+\lambda_{m}L_{m}^{n}+\lambda_{ms}L_{ms}^{n}+\lambda_{f}L_{f}^{n}\right),$$

where $\lambda_{s}$, $\lambda_{m}$, $\lambda_{ms}$, $\lambda_{f}$ are the weight of depth smoothness loss, weight of mask regularization term, weight of mask smoothness loss and weight of feature pyramid matching loss respectively. The settings for them are described in Section 4.1. The total loss is applied on four scales to combat the problem of holes caused by gradient locality [10], and *n* indexes are considered over different depth map scales. The photometric error, the OAM and the FPML elaborated in Section 3.2, Section 3.3 and Section 3.4 respectively.

### 3.2. Photometric Error and Smooth Loss

Under the assumption of surface Lambertian and static rigid scenes, the brightness of the same object under different views should be consistent. Therefore, the current frame ${\hat{I}}_{f}$ synthesized by the depth, camera egomotion and adjacent frame images should be similar to the current frame $I_{t}$. We construct a robust photometric error loss function as follows,

(3) $$L_{p}=\sum_{f\in \{t-1,t+1\}}V_{f}M_{f}\delta \left(I_{t},{\hat{I}}_{f}\right),$$

where $\delta (I_{t},I_{f})$ represents the difference between the current frame and the reconstructed frame, $\delta \left(I_{t},I_{f}\right)=\alpha \frac{1-SSIM(I_{t},I_{f})}{2}+\left(1-\alpha \right){\left\|I_{t}-I_{f}\right\|}_{1}$; SSIM is structural similarity index [20]; $M_{f}$ and $V_{f}$ are the consistent mask and occlusion mask respectively, which are defined in Section 3.3.

In order to make the depth smooth and the edge of it sharp, we also use the following image gradient [9] based depth smoothness loss function,

(4) $$L_{s}=|{▽}_{x}D_{t}|e^{-|{▽}_{x}I_{t}|}+\left|{▽}_{y}D_{t}\right|e^{-|{▽}_{y}I_{t}|},$$

where ${▽}_{x}$ and ${▽}_{y}$ represent the gradients in X and Y directions, respectively.

### 3.3. Occlusion-Aware Mask

**Figure 2:**
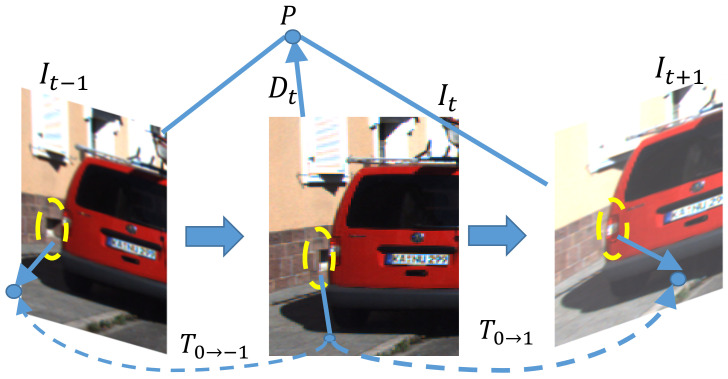
Example of occlusion.

Photometric consistency assumes that the scene is static and the objects are nonoccluded. However, dynamic objects and occlusion usually occur in real scenes. As shown in Figure 2, the pixels in the yellow dash area are visible in the past frame $I_{t-1}$ and current frame $I_{t}$ but blocked by the vehicle in the next frame $I_{t+1}$. If the network predicts the correct depth of the pixels in the yellow dashed area in current frame, then the corresponding occluded area in the next frame does not match the the current frame. This condition results in the large photometric error. The average photometric error is affected by occlusion. Occlusion often occurs at the edge of the object and the inferred incorrect depth. Thus, we propose a multiframe formulation to train a network for predicting occlusions.

We assume a object is visible in the current frame. Depending on whether the corresponding pixel on adjacent frames is visible, there are four cases of the corresponding pixel as follows: visible in all adjacent frames, occluded in all adjacent frames, occluded in the past or occluded in the future. The case that a pixel occluded in all adjacent frames rarely occurs in practice is discarded.

The input of MaskNet is the current and adjacent frames $I=[I_{k},I_{f}]$, and the output is the consistent masks $M_{f}$ corresponding to the reconstructed frames ${\hat{I}}_{f}$. Each element on the consistent mask indicates probability that the pixel satisfies photometric consistency assumption. If pixel $p_{t}$ satisfies photometric consistency assumption in the adjacent frames, we have $I_{t}\left(p_{t}\right)={\hat{I}}_{f}\left(p_{t}\right),f\in \left\{t-1,t+1\right\}$, and $M_{t-1}\left(p_{t}\right)=M_{t+1}\left(p_{t}\right)$. When occlusion only occurs in the past frame, we have $\|I_{t}\left(p_{t}\right)-{\hat{I}}_{t-1}\left(p_{t}\right){\|}_{1}>{\left\|I_{t}\left(p_{t}\right)-{\hat{I}}_{t+1}\left(p_{t}\right)\right\|}_{1}$ and $M_{t-1}\left(p_{t}\right)<M_{t+1}\left(p_{t}\right)$. Otherwise, we have $M_{t-1}\left(p_{t}\right)>M_{t+1}\left(p_{t}\right)$. We extract occlusion masks $V_{t-1}$ and $V_{t+1}$ from consistent masks $M_{t-1}$ and $M_{t+1}$ to indicate whether pixels are visible on the adjacent frames. When $M_{t-1}\left(p_{t}\right)>M_{t+1}\left(p_{t}\right)$, $p_{t}$ is more likely to be visible in the past frame than in the future; as a result, $V_{t-1}\left(p_{t}\right)=1,V_{t+1}\left(p_{t}\right)=0$. If $M_{t-1}\left(p_{t}\right)=M_{t+1}\left(p_{t}\right)$, there are two situations; if $M_{t-1}\left(p_{t}\right)$ and $M_{t+1}\left(p_{t}\right)$ tend to zero, there may be dynamic objects in the adjacent frames, and if they tend to one, there are no dynamic objects. For occlusion, we let $V_{t-1}\left(p_{t}\right)=V_{t+1}\left(p_{t}\right)=0.5$, it means $p_{t}$ is visible in all adjacent frames.

Similar to SfmLearner [10], we add a regularization term of mask, that is,

(5) $$L_{m}=-e^{\beta \|M_{t-1}-M_{t+1}{\|}_{1}}\left(\log M_{t-1}+\log M_{t+1}\right).$$

In other words, the loss prevents the mask to always be zero, since most points in the scene meet the photometric consistent. We also introduce the smoothing loss of the mask to ensure that the pixels in the neighborhood have the similar state, that is,

(6) $$L_{ms}=\sum_{f\in \{t-1,t+1\}}|{▽}_{x}M_{f}|e^{-|{▽}_{x}I_{t}|}+\left|{▽}_{y}M_{f}\right|e^{-|{▽}_{y}I_{t}|}.$$

### 3.4. Feature Pyramid Matching Loss

**Figure 3:**
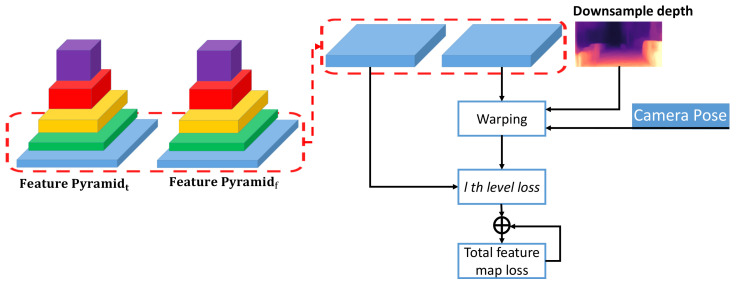
Feature pyramid matching error of the current frame and adjacent frame. Feature pyramid is constructed by different scale feature maps. Adjacent feature maps warped using the downsampled depth and camera pose computes a matching error.

To consider both global high-level and local detailed information, we extract feature pyramid from images and construct FPML for reducing the effect of brightness change and non-Lambertian surface. Figure 3 summarizes the key processes of FPML, which consists of feature pyramid and matching error. Given current image $I_{t}$ and adjacent frames $I_{f}$, we generate L levels pyramid feature, *l*th current feature map $c_{t}^{l}$ and *l*th adjacent feature map $c_{f}^{l}$. Specifically, current image and adjacent frames are input to DepthNet, and the layers of conventional filters output the different scale feature maps to construct the feature pyramid. The encoder module of DepthNet generates a feature pyramid with L=5 layers, and the numbers of feature channels are 64, 64, 128, 256 and 512. FPML makes use of the features generated in the encoder and therefore causes a minimal overhead. We synthesize the current frame feature map by warping ${\hat{c}}_{f}^{l}=g\left(D_{t}^{l},T_{t\to f},c_{f}^{l}\right)$ according to the feature map $c_{f}^{l}$ generated by adjacent frames, downsampled depth map $D_{t}^{l}$ of current frame and camera egomotion $T_{t\to f}$. The resolution of $D_{t}^{l}$ is same as that of *l*th feature map $c_{f}^{l}$. The corresponding feature of the same object in different frames is similar regardless of brightness changes, occlusion and dynamic objects. Thus, we define cosine similarity loss between *l*th feature maps as follows,

(7) $$L_{f}^{l}=1-\frac{{c_{t}^{l}}^{T}{\hat{c}}_{f}^{l}}{\|c_{t}^{l}\left\|\right\|{\hat{c}}_{f}^{l}\|}.$$

The total FPML function is

(8) $$L_{f}=\sum_{f\in \{t-1,t+1\}}\sum_{l\in \{0,1,2,3,4\}}L_{f}^{l}.$$

### 3.5. Network Architecture

**DepthNet and MaskNet**

The DepthNet and MaskNet we proposed based on encoder-decoder architecture, in which the decoder part can share the shallow information of the encoder part through skip connections.

The encoder part adopts the standard ResNet18 [33], which contains 11M parameters and uses the weights pretrained on ImageNet as the initial parameters. The difference of the encoder parts between the DepthNet and MaskNet is the number of input images. The first convolution layer parameter of the DepthNet is 3×64×3×3. The first convolution layer parameter of the MaskNet is set as 9×64×3×3 for adapting to the input images.

In the decoder modules, ELU [34] is adopted as all nonlinear activation functions; five times of upsampling can obtain the feature map with the same resolution of input image, and the upsampling parts use bilinear interpolation. Like SfmLearner [10], the decoder output layer of the DepthNet is activated by sigmoid and converted into a non-negative reasonable depth map. The process is formulated as $D=\frac{1}{a*sigmoid(x)+b}$, where a=10 and b=0.1. The MaskNet uses sigmoid activation to output two channels mask images corresponding to the adjacent frames. Similar to Godard et al. [9] in border filling, we use reflection padding instead of zero padding, which can reduce the border artifacts of the depth map.

**MotionNet**

The input of MotionNet contains RGB images of the current frame and adjacent frames, and the outputs are camera poses of the current frame and adjacent frames. MotionNet consists of a ResNet18 and four convolution layers. The parameter of ResNet18 input layer is 9×64×3×3, and the weights pretrained in ImageNet are also used as initial parameters. All activation functions use RELU, except for the last output layer. The output of the last layer is two channels 6D vector $\phi \in R^{2*6}$, including a 3D rotating Euler angle and a 3D position.

## 4. Experiments

In this section, we compare results of our method with existing state-of-the-art approaches on depth and camera egomotion estimation.

### 4.1. Experimental Settings

**Implementation details**

Our models are implemented with PyTorch [35] and trained for 20 epochs. We set the initial value of loss weights based on experience and other similar papers [9,10,11], and then tune them with a sampled validation set from training images. In our entire training process, we set weight of depth smoothness loss $\lambda_{s}=10^{-3}$, weight of mask regularization term $\lambda_{m}=0.12$, weight of mask smooth loss $\lambda_{ms}=10^{-3}$ and weight of FPML $\lambda_{f}=0.01$. During training, we use the Adam optimizer [36] with $\beta_{1}=0.9$, $\beta_{2}=0.999$. We also set the learning rate of the first 15 epochs to $10^{-4}$, and then to $10^{-5}$ and mini-batch size of 12. All the images in experiments are from KITTI monocular image sequences.

**KITTI dataset**

We use the KITTI [37] dataset as the main dataset for training and testing. In previous works [7,8,9,10,11,12,13,14,15,28,29], KITTI is often used to evaluate performance on depth and egomotion. The KITTI dataset contains images collected by four cameras (two grayscale and two RGB), as well as point cloud collected by a Velodyne HDL-64E laser scanner and pose collected by GPS/IMU. The KITTI dataset provides videos from 200 different scenes, including city streets, roads and campus, etc. During the training, 156 image sequences without test scenes are used, and the left and right images are treated independently. Furthermore, we follow SfmLearner’s preprocessing to remove static frames [10]. A total of 40,109 are obtained for training and 4431 for validation. We choose the Eigen split [7] for depth testing. The Eigen split consists of 697 images, where the depth ground truth is obtained by projecting the Velodyne laser scanned points into the image plane. During the training, the input images are resized to resolution of 640×192, and the camera intrinsic matrix are known. During the validating and testing, the input images use the resolution of 1216×352. KITTI Odometry dataset has 00–10 sequences with pose labels. We follow SfmLearner [10], and split sequences 00–08 for training and 09–10 for testing.

**Evaluation metric**

We use the depth evaluation metric of Eigen et al. [7]. The explanation of each metric adopted in our evaluation is specified in Table 1, where $D^{*}$ and *D* represent the ground truth and estimated depths respectively.

We use absolute trajectory error (ATE) [38] to evaluate camera motion. ATE first aligns the estimated camera motion with the ground truth pose and then evaluates the relative error of camera pose.

### 4.2. Depth Estimation Results

Quantitative comparison results of our method and previous methods are shown in Table 2. The mono column denotes whether stereo camera is used, M means monocular, S indicates stereo. The supervised column denotes whether additional supervised information is used. In the first row, the upward arrow ↑ indicates higher is better, the downward arrow ↓ means lower is better. The best results in each category are printed in bold. Following other traditional methods [7,10], we limit the maximum depth to 80 m. Depth estimation in an unsupervised manner from monocular videos obtains related depth. So, we multiply the estimated depth by the median scale factor $s=median\left(D^{*}\right)/median\left(D\right)$ [10] for comparison with absolute depth generated from stereo camera or supervised methods. Our method outperforms previous supervised methods [7,39] and unsupervised methods [6,9,10,11,13,14,15,16,17,40,41]. Compared with these works mentioned above, our method reduces Abs Rel by 0.017–0.088, Sq Rel by −0.039–0.700, RMSE by 0.187–1.752 and RMSE log by 0.015–0.083. Compared with Struct2depth(M) [13], which uses motion model, our result is 0.021 better than Struct2depth(M) in terms of Abs Rel, 0.187 better than that in terms of RMSE, 0.015 better than that in terms of RMSE log, 0.052 better than that in terms of δ<1.25, and 0.009 better than that in terms of $\delta <1.25^{2}$, except in Sq Rel and $\delta <1.25^{3}$. It is also worth noting that on the metric of Abs Rel, our method outperforms other methods. This metric measures the ratio of prediction error over the ground truth value and can be used to compare the reliability of different depth measurement results. The good performance under this metric indicates that our method produces consistent depth at long and short distances.

Our DepthNet and MotionNet are the same as those of methods in literature [10,13], so the network inference time is also the same. Our test results show that for predicting depth it takes 3.972 s to load model and initialize, 0.020 s for network inference and 0.003 s for postprocessing. For predicting camera egomotion, it takes 4.132 s to load model and initialize, 0.005 s for network inference and 0.002 s for postprocessing.

**Figure 4:**
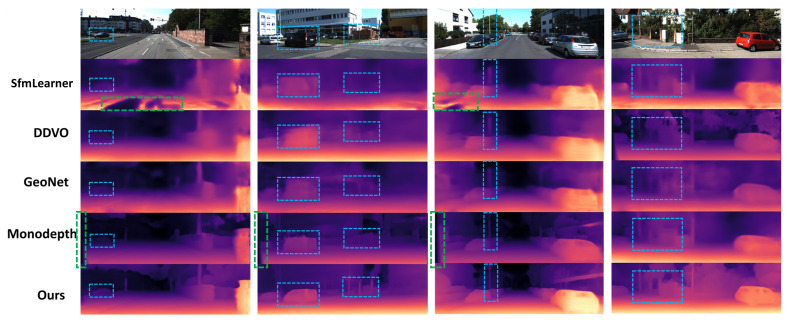
Qualitative KITTI results. Our method is compared with the results of SfmLearner [10], DDVO [17], GeoNet [11] and Monodepth [9]. The higher intensity of red in the picture, the closer the distance. The results in the blue dashed boxes are the areas we focus on. The results in the green dashed boxes are “holes” in the depth maps.

**Figure 5:**
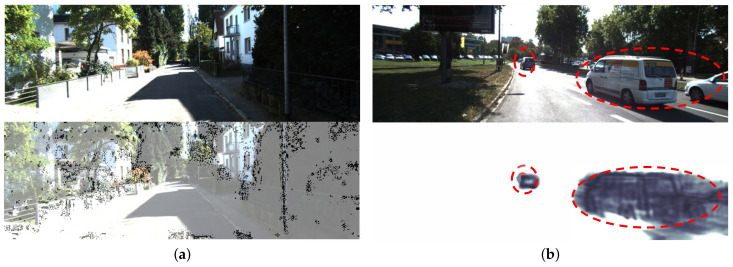
(**a**) occlusion mask (**b**) moving objects mask.

In Figure 4, our experimental results are compared with Sfmlearner, DDVO, GeoNet and Monodepth methods. The first line is the original image and the following is the depth maps generated by each method. The higher intensity of red in the depth map, the closer the distance. The blue boxes in Figure 4 are the areas we focus on, which include objects with broad shape as well as thin objects. Compared with other methods, the depth maps produced by our method are clearer and the edges are sharper in both cases. In the blue boxes of first column images, there is a farther vehicle. DDVO, GeoNet and Monodepth do not estimate its depth, but our method estimates its depth accurately. The boxes in the second and third columns of images include slender pillars, and the boundaries of these objects estimated by other methods are blurry. The green dotted boxes in the image indicate obvious defects in other baselines. We can see that our models generate higher quality outputs and do not produce “holes” in the depth maps. There are holes in the ground in the results of the SfmLearner, which may lead to autonomous vehicles misjudge the passing area. In the results of Monodepth, the depth estimation of the edge area of the image is wrong, which may be caused by the lack of covisible areas in the edge of the stereo images. As shown in Figure 5a, a black region obtained from OAM indicates a possible occlusion in the previous frame. Figure 5b obviously indicates dynamic objects in the scene learned from the MaskNet.

### 4.3. Camera Pose Estimation Results

Our method is compared not only with the traditional visual SLAM method [42] but also with other deep learning methods [10,29]. The quantitative evaluation of camera egomotion estimation is shown in Table 3. Table 3 shows that our camera egomotion results exceed unsupervised learning method monodepth2 [29] and SfmLearner [10] in 09 and 10 sequences in terms of the ATE [38]. We compare our egomotion estimation with two variants of monocular ORB-SLAM [42]. The results show that our method has an advantage over ORB-SLAM(short), which runs on five-frame snippets. Our results are not as good as ORB-SLAM(full) because ORB-SLAM(full) is a complete SLAM system including loop closure and relocalization, which uses all images in the sequence.

### 4.4. Ablation Study

We measure the impact of each contribution on performance and show the results of ablation study in Table 4 to understand which part of our method contributes to the performance. In Table 4, the baseline model following recent works [10,11] does not contain any of our contributions; +F represents the contribution of FPML; +OM indicates the contribution of OAM. Comparing with the baseline, the performance is improved by adding the FPML or OAM. In the main metric Abs Rel, the contribution of FPML is 0.01 better than that of the baseline. Moreover, the contribution of OAM is 0.013 better than that of the baseline. The combination of these contributions improves performance by 0.02 better than the baseline in terms of Abs Rel.

## 5. Conclusions

We propose an unsupervised learning framework that achieves monocular depth and egomotion estimation via FPML and OAM. The introduced FPML captures the local and global information and reduces the influence of brightness variation and non-Lambertian surface. In addition, the proposed OAM predicts not only dynamic objects but also occluded pixels in an innovative manner according to change of masks. As a result, FPML and OAM address the problem of photometric inconsistency and improve accuracy of depth and camera pose estimation. On the KITTI dataset, our results are better than the state-of-the-art unsupervised methods and even some supervised methods, both qualitatively and quantitatively. Especially, compared with previous methods, our method reduces Abs Rel by 0.017–0.088, which is the most important metric in the literature.

In our future works, we will estimate the 3D motion of the dynamic rigid object in the image to help the robot better understand the 3D environment. Furthermore, the camera and LiDAR information will also be fused to achieve real-time accurate depth estimation, which is used for localization and mapping.

## Figures and Tables

**Table 1:** Depth evaluation metric.

Abs Rel: $\frac{1}{\left|T\right|}\sum_{D\in T}\frac{|D-D^{*}|}{D^{*}}$
Sq Rel: $\frac{1}{\left|T\right|}\sum_{D\in T}\frac{|D-D^{*}{|}^{2}}{D^{*}}$
RMSE log: $\sqrt{\frac{1}{\left|T\right|}\sum_{D\in T}{\left|\log\frac{D}{D^{*}}\right|}^{2}}$
RMSE:$\sqrt{\frac{1}{\left|T\right|}\sum_{D\in T}{\left|D-D^{*}\right|}^{2}}$
$\delta_{t}$:% of $D\in T\max(\frac{D^{*}}{D},\frac{D}{D^{*}})<t$

**Table 2:** Depth estimation quantitative results on Eigen [7] split of KITTI raw dataset [37], capped at 80 m. These methods are all trained on KITTI raw dataset. The camera column denotes whether stereo camera is used, M means monocular, S indicates stereo. The supervised column denotes whether using additional supervised information. In the first row, the upward arrow ↑ indicates higher is better, the downward arrow ↓ means lower is better. Best results in each category are in bold.

Method	Supervied	Camera	Abs Rel↓	Sq Rel↓	RMSE↓	RMSE log↓	δ<1.25↑	$\delta <1.25^{2}$↑	$\delta <1.25^{3}$↑
Eigen [7]	Depth	M	0.203	1.548	6.307	0.282	0.702	0.890	0.890
Liu [39]	Depth	M	0.201	1.584	6.471	0.273	0.680	0.898	0.967
SfmLearner [10]	-	M	0.208	1.768	6.856	0.283	0.678	0.885	0.957
Yang [15]	-	M	0.182	1.481	6.501	0.267	0.725	0.906	0.963
Vid2depth [16]	-	M	0.163	1.240	6.220	0.250	0.762	0.916	0.968
LEGO [14]	-	M	0.162	1.352	6.276	0.252	0.783	0.921	0.969
GeoNet [11]	-	M	0.155	1.296	5.857	0.233	0.793	0.931	0.973
DDVO [17]	-	M	0.151	1.257	5.583	0.228	0.810	0.936	0.974
Monodepth [9]	Pose	S	0.148	1.344	5.927	0.247	0.803	0.922	0.964
CC [40]	-	M	0.148	1.149	5.464	0.226	0.815	0.935	0.973
EPC++ [41]	-	M	0.141	1.029	5.350	0.216	0.816	0.941	0.976
Struct2depth(M) [13]	-	M	0.141	**1.026**	5.291	0.215	0.816	0.945	**0.979**
SC-SfmLearner [6]	-	M	0.137	1.089	5.439	0.217	0.830	0.942	0.975
**Ours**	-	M	**0.120**	1.065	**5.104**	**0.200**	**0.868**	**0.954**	0.978

**Table 3:** Absolute Trajectory Error (ATE) on the KITTI Odometry sequences 09 and 10 (lower is better).

Method	Seq.09	Seq.10
ORB-SLAM(full) [42]	0.014±0.008	0.012±0.011
Mean Odometry	0.032±0.026	0.028±0.023
ORB-SLAM(short)	0.064±0.141	0.064±0.130
Monodepth2 [29]	0.023±0.013	0.018±0.014
SfmLearner [10]	0.021±0.017	0.020±0.015
**Ours**	0.019±0.009	0.013±0.010

**Table 4:** Ablation studies on FPML and OAM. +F represents the contribution of FPML. +OM indicates the contribution of OAM. Each of our contributions improves performance.

Method	Abs Rel	Sq Rel	RMSE	RMSE log	δ<1.25	$\delta <1.25^{2}$	$\delta <1.25^{3}$
Baseline	0.140	1.610	5.512	0.223	0.852	0.946	0.973
+F	0.130	0.974	5.197	0.208	0.840	0.948	0.979
+OM	0.127	0.957	5.163	0.202	0.852	0.953	0.980
+OM+F	0.120	1.065	5.104	0.200	0.868	0.954	0.978

## Data Availability

Not applicable.

## Abbreviations

The following abbreviations are used in this manuscript:

FPML	Feature Pyramid Matching Loss
OAM	Occlusion-Aware Mask
Abs Rel	Absolute Relative Error
SAD	Sum of Absolute Differences
SSIM	Structural Similarity
SAD	Sum of Absolute Differences
SOTA	State-Of-The-Art
CNN	Convolutional Neural Network
Mask R-CNN	Mask Region—Convolutional Neural Networks
Sq Rel	Squared Relative Error
RMSE	Root Mean Squard Error
ELU	exponential linear unit
RELU	Rectified Linear Unit
GPS	Global Position System
IMU	Inertial Measurement Unit
ATE	Absolute Trajectory Error
